# Effect of Quercetin on PC12 Alzheimer's Disease Cell Model Induced by A*β*_25-35_ and Its Mechanism Based on Sirtuin1/Nrf2/HO-1 Pathway

**DOI:** 10.1155/2020/8210578

**Published:** 2020-04-27

**Authors:** Xinjun Yu, Yicai Li, Xiaohua Mu

**Affiliations:** ^1^Department of Dizziness, Affiliated Hospital of Weifang Medical College, Weifang 261031, China; ^2^Department of Neurology, Affiliated Hospital of Weifang Medical College, Weifang 261031, China; ^3^Department of Neurology, Weifang Brain Hospital, Weifang 261021, China

## Abstract

**Objective:**

This study is aimed at studying the effect of quercetin on the Alzheimer disease cell model induced by A*β*_25-35_ in PC12 cells and its mechanism of action.

**Methods:**

The AD cell model was established by A*β*_25-35_. Quercetin was used at different concentrations (0, 10, 20, 40, and 80 *μ*mol/L). The morphology of cells was observed, and the effect on cell survival rate was detected by the MTT method. Cell proliferation was detected by the SRB method. The contents of LDH, SOD, MDA, GSH-Px, AChE, CAT, and T-AOC were detected by kits. The expression of sirtuin1/Nrf2/HO-1 was detected by RT-qPCR and Western blot.

**Results:**

PC12 cells in the control group grew quickly and adhered well to the wall, most of which had extended long axons and easily grew into clusters. In the model group, cells were significantly damaged and the number of cells was significantly reduced. It was found that PC12 cells were swollen, rounded, protruding, and retracting, with reduced adherent function and floating phenomenon. Quercetin could increase the survival rate and proliferation rate of PC12 cells; reduce the levels of LDH, AChE, MDA, and HO-1 protein; and increase the levels of SOD, GSH-Px, CAT, T-AOC, sirtuin1, and Nrf2 protein.

**Conclusion:**

Quercetin can increase the survival rate of PC12 injured by A*β*_25-35_, promote cell proliferation, and antagonize the toxicity of A*β*; it also has certain neuroprotective effects. Therefore, quercetin is expected to become a drug for the treatment of AD.

## 1. Introduction

Alzheimer's disease (AD) is a neurodegenerative disease that accounts for 50% of dementia cases worldwide [1]. The incidence of AD is increasing year by year [2]. It seriously affects the quality of life. It has become one of the most serious problems in the field of geriatrics. At present, the specific pathogenesis of AD is not clear, but studies have found that *β*-amyloid (A*β*) deposition, neurofibrillar tangles, and neuronal loss are the main characteristics of AD, which are closely related to the cognitive impairment of patients [3–5]. It has been shown that the deposition of A*β*_25-35_ has a direct toxic effect on neurons, and it can also enhance the sensitivity of neurons to harmful factors such as free radicals and oxidative stress, thus leading to neuronal apoptosis [6]. Therefore, the inhibition of neuronal apoptosis induced by A*β* protein provides a feasible method for the prevention and treatment of AD.

At present, the main drugs used in the treatment of AD are cholinesterase inhibitors, glutamate receptor antagonists, antioxidants, and neuroprotective agents, but they can only partially alleviate the symptoms of AD. Traditional Chinese medicine may have potential advantages and characteristics in the prevention and treatment of AD. Quercetin is a flavonoid found in many vascular plants, such as onions, tea, coffee, and berries [7]. It has strong pharmacological effects such as anticancer [8], antioxidation [9], lowering blood pressure [10], antiradiation [11], neuroprotection [12], and inhibiting platelet aggregation and capillary permeability [13]. In recent years, it has been reported that quercetin can inhibit the proliferation of tumor cells [14–16]. In addition, more and more attention has been paid to the potential of quercetin in the treatment of neurodegenerative diseases [17–19]. However, the underlying mechanism is not clear.

AD could be mimicked *in vitro* by treating neuron cells with A*β*_25-35_ [6, 20], as evidenced by increased oxidative stress and mitochondrial damage. Herein, an *in vitro* AD model induced by A*β*_25-35_ was established in PC12 cells. The purpose of this study was to explore the effect of quercetin on AD by using this model. The possible role of the sirtuin1/nuclear factor E2-related factor 2 (Nrf2)/heme oxygenase-1 (HO-1) pathway in the effect of quercetin was also analyzed and discussed. Our findings may provide theoretical basis for the use of quercetin in the treatment of AD.

## 2. Materials and Methods

### 2.1. Reagents

Rat adrenal pheochromocytoma cells (PC12 cells; highly differentiated type) were purchased from the Cell Center of Shanghai Academy of Life Sciences, Chinese Academy of Sciences. A*β*_25-35_ (purity ≥ 95%, no. Y0044) was from Beijing Biosynthesis Biotechnology Co., Ltd. (China). DMEM, streptomycin, trypsin, and fetal bovine serum were from HyClone Laboratories Inc. (Logan, Utah, USA). MTT was from Sigma-Aldrich (Merck KGaA, Darmstadt, Germany). A lactate dehydrogenase (LDH) kit was purchased from Beyotime Biotechnology Co., Ltd. (Shanghai, China). Superoxide dismutase (SOD), malondialdehyde (MDA), myeloperoxidase (MPO), and glutathione peroxidase (GSH-Px), as well as catalase (CAT), total antioxidant capacity (T-AOC), and acetylcholinesterase (AChE) kits were purchased from Nanjing Jiancheng Bioengineering Institute (Nanjing, China). The primary antibodies against sirtuin1 (Cat. No. 13161-1-AP), total Nrf2 (Cat. No. 16396-1-AP), nuclear Nrf2 (Cat. No. 66504-1-Ig), HO-1 (Cat. No. 66743-1-Ig), *β*-actin (Cat. No. 60008-1-Ig), and PCNA (Cat. No. 60097-1-Ig) were from Proteintech Co., Ltd. (Chicago, USA). HRP-labeled goat anti-mouse IgG antibody was purchased from Santa Cruz Biotechnology, Inc. (Cat. No. sc-2005; Santa Cruz, California, USA). HRP-labeled goat-rabbit IgG antibody was from Proteintech Co., Ltd. (Cat. No. 10285-1-AP; Chicago, USA).

### 2.2. Cell Culture

PC12 cells were cultured in DMEM+10% fetal bovine serum+streptomycin at 37°C, 5% CO_2_, and subcultured every 3 to 4 days. The growth of PC12 cells was observed by an inverted microscope.

### 2.3. Establishment of AD Cell Model

The AD cell model was established as previously described [20]. Briefly, PC12 cells were seeded in a 96-well plate at a concentration of 10^4^/mL. A*β*_25-35_ at concentrations of 0, 5, 10, 20, 30, and 40 *μ*mol/L was added into cells and incubated for 24 h. The cell survival rate was measured by the MTT method. The concentration of A*β*_25-35_, which reduced the cell survival by 50% to 60%, was selected to establish the AD cell model.

### 2.4. Experimental Grouping

In the control group, cells were not treated with any drugs. In the model group, cells were treated with 20 *μ*mol/L A*β*_25-35_ for 24 h to establish the AD cell model. In the quercetin group, cells were pretreated with quercetin at 10, 20, 40, and 80 *μ*mol/L for 24 h, 48 h, and 72 h, and then with 20 *μ*mol/L A*β*_25-35_ for 24 h.

### 2.5. MTT Assay

PC12 cells were seeded in a 96-well plate at a concentration of 10^4^/mL and cultured for 24 h. To detect the effect of A*β*_25-35_, cells were treated with A*β*_25-35_ at concentrations of 0, 5, 10, 20, 30, and 40 *μ*mol/L for 24 h. To detect the effect of quercetin, cells were treated and grouped as described above. Then, 20 *μ*L of MTT was added and incubated for 4 h in the dark. After that, 150 *μ*L of DMSO was added and the plate was oscillated on a shaking table for 10 min until the blue crystals were completely dissolved. The absorbance of each well at a wavelength of 570 nm was detected by an automatic microplate analyzer. The proliferation rate of PC12 cells was calculated according to the following formula: cell survival rate (%) = experimental group OD_570_/control group OD_570_ × 100%.

### 2.6. Determination of LDH Content

PC12 cells were seeded in a 96-well plate at a concentration of 10^4^/mL. They were treated and grouped as mentioned before. The LDH level was determined by an LDH kit according to the instructions provided. The absorbance (OD value) was determined by an enzyme-labeling instrument at a wavelength of 450 nm. LDH activity = (ODu − ODc)/(ODs − ODb) × Cs × *N* × 1000; annotation: ODu is the absorbance value of the measuring tube; ODc is the absorbance value of a blank tube; ODs is the absorbance value of a standard tube; ODb is the absorbance value of the control tube; Cs is standard concentration (2 mmol/L); and *N* is the dilution multiple of the sample before the test.

### 2.7. Cell Proliferation Assay

PC12 cells were treated and grouped as mentioned before. Then, 50% trichloroacetic acid was added to each well and incubated at 4°C for 1 h. After drying, 50 *μ*L of 0.4% SRB solution was added and incubated for 15 min. After that, the wells were rinsed with a small amount of 1% acetic acid solution for 2~3 times. After drying, 100 *μ*L of 10 mmol/L Tris alkali solution was added to dissolve the crystals in each well. The absorption value (OD) at the wavelength of 540 nm was measured with a microplate analyzer (HBS-1096B; Beijing Hezhong Bopp Technology Development Co., Ltd., China).

### 2.8. ELISA

After treatment, PC12 cells of each group were collected and subjected to cell lysis. After centrifugation at 14,000 g for 10 min, the supernatant was collected. The ELISA method was used to detect the changes of the oxidative stress index for SOD, GSH-Px, CAT, and T-AOC according to the kit instructions. AChE and MDA in the supernatant were also measured with corresponding kits according to the instructions.

### 2.9. Reverse Transcription-Quantitative Polymerase Chain Reaction (RT-qPCR)

Total RNA of each group was extracted and then reverse transcribed into cDNA. The mRNA levels of sirtuin1, Nrf2, and HO-1 were detected with RT-qPCR. The reaction conditions of RT-qPCR were as follows: predenaturation at 94°C for 5 min and 45 cycles of 95°C for 5 s and 60°C for 30 s. The gel density was scanned by an image analyzer, and the relative content of mRNA was obtained. The sequence of RT-PCR reaction primers is listed in Table 1.

### 2.10. Western Blot Analysis

PC12 cells were subjected to lysis with RIPA buffer after treatment. Protein concentration was determined by a BCA kit, and 100 *μ*g of total protein was separated by 10%SDS-PAGE electrophoresis. The proteins were then transferred to a PVDF membrane. The membrane was blocked with 5% milk for 1 h at room temperature, and the primary antibodies were incubated at 4°C overnight. After washing, the secondary antibodies were added and incubated at room temperature for 1 h. Chemiluminescence was used for color development, and image analysis was performed with Quantity One software (Media Cybernetics, Inc., Rockville, MD, USA).

### 2.11. Statistical Analysis

Each experiment was repeated 3 times. Data were expressed by mean ± standard deviation, and one-way ANOVA (*F* test) was performed by SPSS21.0 statistical software. *P* < 0.05 indicates significant difference.

## 3. Results

### 3.1. Effect of A*β*_25-35_ on the Survival Rate of PC12 Cells

**Figure 1:**
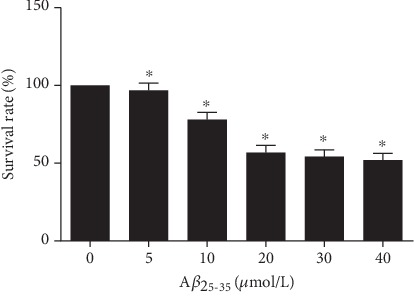
Effect of A*β*_25-35_ on the survival rate of PC12 cells. PC12 cells were treated with A*β*_25-35_ concentrations of 0, 5, 10, 20, 30, and 40 *μ*mol/L for 24 h. Cell survival was analyzed with the MTT assay. Note: ^∗^*P* < 0.05, compared with the 0 *μ*mol/L A*β*_25-35_.

To detect the effect of A*β*_25-35_ on cell survival, PC12 cells were treated with different concentrations of A*β*_25-35_. Cell survival was assessed with the MTT assay. Generally, in the untreated group, PC12 cells grew quickly and adhered to the well wall. Most of them had stretched-out long axons and grew easily in clusters. After treatment with A*β*_25-35_, the cells were obviously damaged and the cell number obviously decreased. The cell survival rate decreased along with the increase of A*β*_25-35_ concentration. When the concentration of A*β*_25-35_ increased from 10 *μ*mol/L to 20 *μ*mol/L, the cell survival rate decreased most significantly (*P* < 0.05, Figure 1). The cell survival at 20 *μ*mol/L A*β*_25-35_ was reduced to 50%-60%. Therefore, we chose A*β*_25-35_ at 20 *μ*mol/L to induce an AD cell model.

### 3.2. Effect of Quercetin on Cell Survival

**Figure 2:**
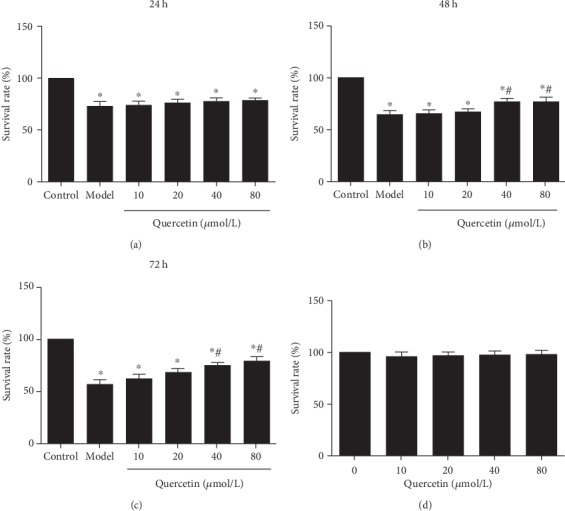
Effect of quercetin on cell survival of PC12 cells. Cells were grouped as follows: the control group (untreated), the model group (cells were treated with 20 *μ*mol/L A*β*_25-35_ for 24 h to establish the AD cell model), and the quercetin group (cells were pretreated with quercetin at 10, 20, 40, and 80 *μ*mol/L for 24 h, 48 h, and 72 h, and then with 20 *μ*mol/L A*β*_25-35_ for 24 h). Cell survival was analyzed with the MTT assay. Cell survival rates at 24 h (a), 48 h (b), and 72 h (c) are shown. (d) Effect of quercetin treatment alone on cell survival. Note: ^∗^*P* < 0.05, compared with the control; ^#^*P* < 0.05, comparison of different concentrations of the quercetin group and the model group.

The MTT assay was used to detect cell survival after treatment with different concentrations of quercetin. As shown in Figure 2, after the treatment with A*β*_25-35_, the AD cell model was established and the survival rate of cells was significantly reduced compared to that of control (*P* < 0.01). Quercetin increased the cell survival rate along with the increased concentration (*P* < 0.05). In addition, the survival rate gradually increased with the extension of treatment time (Figures 2(a)–2(c)). The treatment with quercetin alone at different concentrations did not have a significant effect on the survival rate of PC12 cells (*P* > 0.05, Figure 2(d)). Thus, quercetin could increase the cell survival of the AD cell model.

### 3.3. Effect of Quercetin on LDH Release from Cells

**Figure 3:**
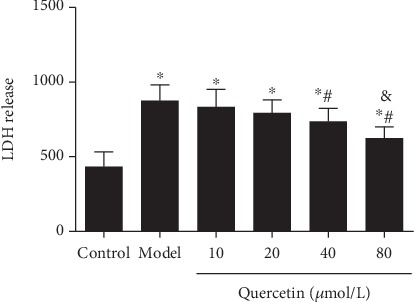
Effect of quercetin on LDH release from cells. Cells were grouped as described above. LDH release was analyzed. Note: ^∗^*P* < 0.05, compared with the control; ^#^*P* < 0.05, comparison of different concentrations of the quercetin group and the model group. ^&^*P* < 0.05, 80 *μ*mol/L quercetin compared with other doses.

The degree of nerve cell injury was proportional to LDH release. After the establishment of the AD cell model by A*β*_25-35_ (model group), LDH release was significantly increased compared to the control group (*P* < 0.01) (Figure 3). However, LDH release was significantly reduced after treatment with different concentrations of quercetin (*P* < 0.05). LDH release was significantly lower at high-dose quercetin (80 *μ*mol/L) than at other doses (*P* < 0.05, Figure 3). This result indicates that quercetin decreases LDH release from the AD cell model.

### 3.4. Effect of Quercetin on Cell Proliferation

**Figure 4:**
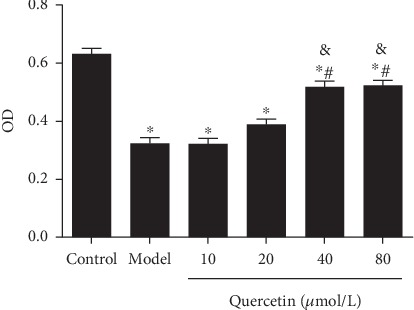
Effect of quercetin on cell proliferation. Cells were grouped as described above. Cell proliferation was detected, and OD540 value was recorded. Note: ^∗^*P* < 0.05, compared with the control; ^#^*P* < 0.05, comparison of different concentrations of the quercetin group and the model group. ^&^*P* < 0.05, 40 *μ*mol/L and 80 *μ*mol/L quercetin compared with other doses.

After the establishment of the AD cell model, the OD value of PC12 cells decreased significantly (*P* < 0.05). After quercetin treatment, the OD value of cells was significantly increased (*P* < 0.05). The OD value of cells with quercetin at high-dose (40 and 80 *μ*mol/L) was significantly higher than those at other doses (*P* < 0.05, Figure 4).

### 3.5. Effect of Quercetin on Antioxidant Capacity of Cells

**Figure 5:**
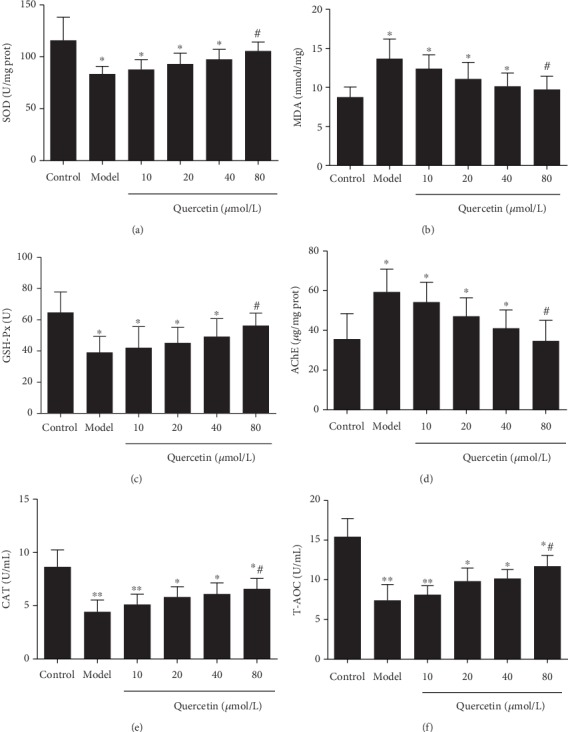
Effect of quercetin on antioxidant capacity of cells. Cells were grouped as described above. The levels of SOD (a), MDA (b), GSH-Px (c), AChE (d), CAT (e), and T-AOC (f) are shown. Note: ^∗^*P* < 0.05 and ^∗∗^*P* < 0.01, compared with the control; ^#^*P* < 0.05, comparison of different concentrations of the quercetin group and the model group.

The antioxidant capacity of cells was further analyzed. Compared with control, the contents of SOD (Figure 5(a)), GSH-Px (Figure 5(c)), CAT (Figure 5(e)), and T-AOC (Figure 5(f)) in AD model cells were significantly reduced (*P* < 0.05), whereas AChE (Figure 5(d)) activity was enhanced (*P* < 0.05) and MDA (Figure 5(b)) level was increased (*P* < 0.01) in AD model cells. Compared with the model group, the levels of SOD (Figure 5(a)), GSH-Px (Figure 5(c)), CAT (Figure 5(e)), and T-AOC (Figure 5(f)) were significantly increased in the quercetin groups (*P* < 0.05). The quercetin groups also had significantly reduced AChE (Figure 5(d)) activity and MDA (Figure 5(b)) levels than the model group (*P* < 0.05). However, there was no dose-dependent effect in antioxidant capacity of PC12 cells between quercetin concentrations (*P* > 0.05).

### 3.6. Effect of Quercetin on Sirtuin1/Nrf2/HO-1 mRNA Expression in Cells

**Figure 6:**
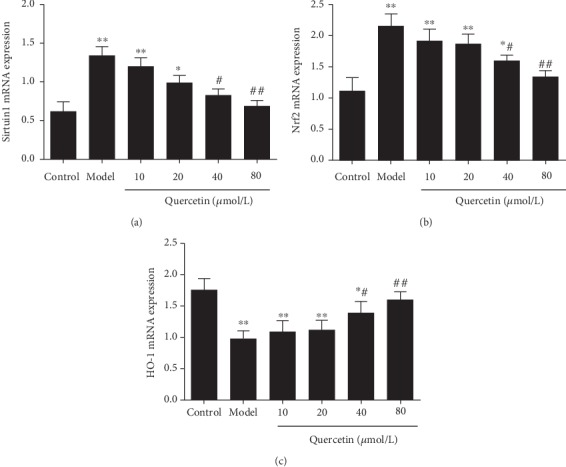
Effect of quercetin on sirtuin1/Nrf2/HO-1 mRNA expression in cells. Cells were grouped as described above. The mRNA level was detected with RT-qPCR. (a) Sirtuin1 mRNA. (b) Nrf2 mRNA. (c) HO-1 mRNA. Note: ^∗^*P* < 0.05 and ^∗∗^*P* < 0.01, compared with the control; ^#^*P* < 0.05 and ^##^*P* < 0.01, comparison of different concentrations of the quercetin group and the model group.

RT-qPCR was conducted to analyze mRNA expression of *sirtuin1*, *Nrf2*, and *HO-1*. The results showed that the AD model had significantly increased expression levels of sirtuin1 (Figure 6(a)) and Nrf2 mRNA (Figure 6(b)) than the control group (*P* < 0.05). In contrast, the expressions of HO-1 mRNA (Figure 6(c)) were significantly decreased in the AD model group than in the control group (*P* < 0.05). Quercetin groups significantly reduced the expression of sirtuin1 (Figure 6(a)) and Nrf2 mRNA (Figure 6(b)) in PC12 cells (*P* < 0.05), while they increased the expression of HO-1 mRNA (Figure 6(c)) (*P* < 0.05).

### 3.7. Effect of Quercetin on the Protein Expression of Sirtuin1/Nrf2/HO-1 in Cells

**Figure 7:**
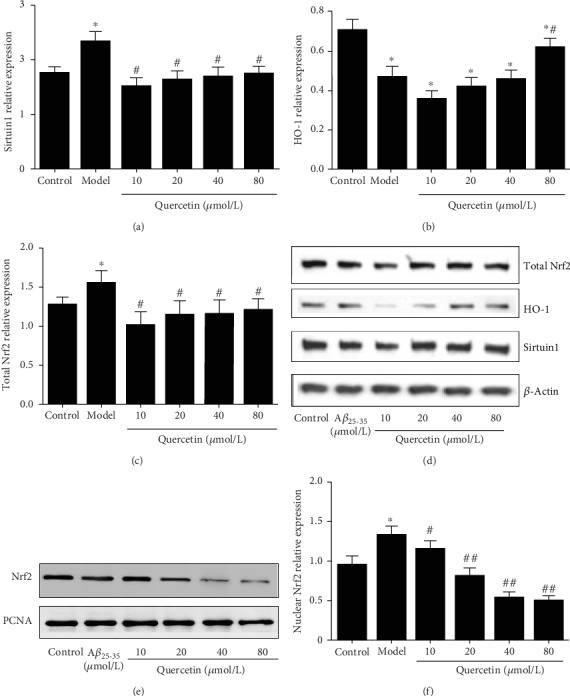
Effect of quercetin on the expression of sirtuin1/Nrf2/HO-1 protein in cells. Cells were grouped as described above. The protein level was detected with Western blot. Relative levels of (a) Sirtuin1 protein, (b) total Nrf2 protein, and (c) HO-1 protein. (d) Representative Western blot images of Sirtuin1, total Nrf2, and HO-1 proteins. (e) Representative Western blot images of nuclear Nrf2. (f) Relative level of nuclear Nrf2. Note: ^∗^*P* < 0.05, compared with the control; ^#^*P* < 0.05 and ^##^*P* < 0.01, comparison of different concentrations of the quercetin group and the model group.

Western blot was used to detect protein expression of sirtuin1, Nrf2, and HO-1. Consistently with the mRNA results, compared with control group, the protein levels of sirtuin1 (Figures 7(a) and 7(d)), total Nrf2 (Figures 7(c) and 7(d)), and nuclear Nrf2 (Figures 7(e) and 7(f)) in PC12 cells were significantly increased (*P* < 0.05), while the protein expression of HO-1 (Figures 7(b) and 7(d)) was significantly decreased in the AD model group (*P* < 0.05). Compared with the AD model group, the protein expression of Sirtuin1 (Figures 7(a) and 7(d)), total Nrf2 (Figures 7(c) and 7(d)), and nuclear Nrf2 (Figures 7(e) and 7(f)) in PC12 cells in the quercetin group was significantly decreased (*P* < 0.05), while the protein expression of HO-1 (Figures 7(b) and 7(d)) was increased in the quercetin group (*P* < 0.05). There was no significant difference among different doses of quercetin (*P* > 0.05).

## 4. Discussion

AD is a representative neurodegenerative disease in learning and memory impairment [1], which can be divided into primary dementia, vascular dementia, and a mixture of the two. Primary dementia is also known as AD. Its main clinical characteristics are cognitive deficits and memory disorders, which may cause aphasia, miscalculation, apraxia, agnosia, and other symptoms, and lead to the impairment of the patient's social life or professional function. So far, the treatment of AD is still very limited. Patients usually need the long-term care of their families and eventually die after a long and painful course of 7 to 10 years. There are about 36 million AD patients in the world, and this number will rise to 100 million by 2050, and the number of AD patients in China has reached nearly 10 million [21]. With the progress of aging, this data will continue to increase [22]. Therefore, it is very urgent to develop an effective treatment for AD.

Quercetin is a kind of polyphenolic flavonoid, the chemical name of which is 3,3′,4′,5,7-pentahydroxyflavone, and it often exists in a variety of food and medicinal plants in the form of glycosides. Quercetin has a strong antioxidant effect, and its chemical structure is similar to that of resveratrol [23]. It has many biological activities, such as antioxidation, anti-inflammation, antitumor, antifibrosis, and protection of cardiocerebral vessels [23]. It is effective in the treatment of cardiovascular disease [24, 25], diabetes [26], inflammation [27], asthma [27], viral infection [29], and cancer prevention [30]. Some studies have shown that quercetin can reduce the degree of cerebral edema after cerebral ischemia-reperfusion, has a strong antioxidant effect, has a direct and indirect scavenging effect on superoxide anion, and prevents the formation of free radicals [31, 32]. It can significantly reduce the production of mitochondrial ROS, enhance the SOD activity of cells, maintain the balance of intracellular oxidation and antioxidant system, reduce the damage of mitochondria, reduce the oxidative stress reaction, increase the value of Δ*ψm*, decrease the content of MDA, alleviate cell injury and apoptosis [33]. The results of this study showed that the contents of SOD, GSH-Px, CAT, and T-AOC were significantly reduced, while AChE activity and MDA levels were significantly increased in AD model cells. After quercetin intervention, the contents of SOD, GSH-Px, CAT, and T-AOC were increased, and AChE activity and MDA levels were decreased. After AD model cells were treated with different concentrations of A*β*_25-35_, the cell activity decreased with the increase of concentration, presenting an A*β*_25-35_ dose-dependent effect. Meanwhile, the cell LDH level increased. After quercetin intervention, the cell activity and cell proliferation rate increased, while the cell LDH level decreased. The effect was more pronounced in the high-dose group. It is suggested that quercetin can increase the activity of AD model cells and inhibit the level of oxidative stress and LDH, thus playing an anti-AD role.

Nrf2, as a defensive transcription factor, is located in the cytoplasm under normal physiological conditions. When stimulated by external oxidative stress, Nrf2 is transferred to the nucleus. Then, it initiates the production of a variety of protective molecules, so as to increase the resistance of cells to external stimulation. The Nrf2 pathway can produce antioxidants and reduce Ca^2+^ overload to protect nerve cells [34]. It is reported that Nrf2 is involved in the antioxidant stress response of cells, and the detection of downstream products such as SOD and MDA can be used to indicate whether the Nrf2 pathway is involved in the process of mitochondrial protection of nerve cells [35]. After the occurrence of AD, the Nrf2 pathway is activated, which can activate glial cells and provide protection for craniocerebral injury. In addition, intracellular Nrf2 can produce an obvious antioxidant effect to reduce the apoptosis of nerve cells. HO-1 is recognized as the most important antioxidant protective protein regulated by Nrf2 [36, 37]. The antioxidant effect of HO-1 is shown in two aspects. First, HO-1 can prevent the free heme from participating in the oxidative stress reaction. Secondly, HO-1 and its catalytic CO and bilirubin have anti-inflammation and antioxidation effects. They can inhibit apoptosis, dilate blood vessels, and improve tissue and cell microcirculation, thus participating in the antioxidant response of tissues and cells in vivo [38]. Therefore, Nrf2/HO-1 play an important role in the oxidative stress response [39]. Sirtuin1 is a NAD^+^-dependent deacetylase in insulin-sensitive tissues, such as liver, skeletal muscle, pancreas, fat, and brain. It can enhance the ability of cells to resist oxidative stress, reduce apoptosis, reduce inflammation, and regulate cell energy metabolism. Activation of the sirtuin1 signaling pathway can improve the cognitive ability of AD mice [40] and regulate neuroinflammatory response [41]. Sirtuin1 may be a promising serum protein marker for early detection of AD [42]. The results of this study showed that the relative levels of sirtuin1 and Nrf2 mRNA and protein expression in AD model cells were significantly increased, while the relative levels of HO-1 mRNA and protein expression were decreased. After quercetin intervention, the relative levels of sirtuin1 and Nrf2 mRNA were decreased, while the relative levels of HO-1 mRNA were increased. Consistently, quercetin had similar effects on the levels of sirtuin1, Nrf2, and HO-1 proteins in PC12 cells. It was suggested that quercetin could increase the survival rate of injured PC12 cells of A*β*_25-35_, promote cell proliferation, combat the toxicity of A*β*_25-35_, and have certain neuroprotective effects against AD, which may be achieved by regulating the sirtuin1/Nrf2/HO-1 pathway.

In conclusion, quercetin can increase the survival rate of A*β*_25-35_-injured PC12 cells, promote cell proliferation, antagonize the toxicity of A*β*, and provide certain neuroprotective effects. Therefore, quercetin is expected to become a drug for the treatment of AD.

## Figures and Tables

**Table 1:** Primer sequences.

	Primer sequence (5′-3′)
*β*-Actin	Forward 5′-ATG GCA ACT GTC CCT GAA CT-3′
	Reverse 5′-GTC ATC ATC CCA CGA GTC AC-3′

Sirtuin1	Forward 5′-CTT GGG ACT GAT TTG AC-3′
	Reverse 5′-CTC TGA ATG ACT CTG GCT TTG-3′

Nrf2	Forward 5′-TGG TGGTTT GCT ACG ACG-3′
	Reverse 5′-CTC CAG AAC TCC AGG CGG-3′

HO-1	Forward 5′-AGT GTG GAG GAT GCC TTG CGA ATG-3′
	Reverse 5′-TGG GCT TTC AAG ACT GGA ACG GTC-3′

## Data Availability

All data are within this manuscript.
